# Neural Stem Cell-Derived Exosomes Revert HFD-Dependent Memory Impairment via CREB-BDNF Signalling

**DOI:** 10.3390/ijms21238994

**Published:** 2020-11-26

**Authors:** Matteo Spinelli, Francesca Natale, Marco Rinaudo, Lucia Leone, Daniele Mezzogori, Salvatore Fusco, Claudio Grassi

**Affiliations:** 1 Department of Neuroscience, Università Cattolica del Sacro Cuore, 00168 Rome, Italy; matteo.spinelli@unicatt.it (M.S.); francesca.natale1@unicatt.it (F.N.); marco.rinaudo@unicatt.it (M.R.); lucia.leone@unicatt.it (L.L.); daniele.mezzogori@unicatt.it (D.M.); claudio.grassi@unicatt.it (C.G.); 2 Fondazione Policlinico Universitario Agostino Gemelli IRCCS, 00168 Rome, Italy

**Keywords:** CREB, synaptic plasticity, high fat diet, exosomes, memory deficits, BDNF, personalized medicine, epigenetics

## Abstract

Overnutrition and metabolic disorders impair cognitive functions through molecular mechanisms still poorly understood. In mice fed with a high fat diet (HFD) we analysed the expression of synaptic plasticity-related genes and the activation of cAMP response element-binding protein (CREB)-brain-derived neurotrophic factor (BDNF)-tropomyosin receptor kinase B (TrkB) signalling. We found that a HFD inhibited both CREB phosphorylation and the expression of a set of CREB target genes in the hippocampus. The intranasal administration of neural stem cell (NSC)-derived exosomes (exo-NSC) epigenetically restored the transcription of Bdnf, nNOS, Sirt1, Egr3, and RelA genes by inducing the recruitment of CREB on their regulatory sequences. Finally, exo-NSC administration rescued both BDNF signalling and memory in HFD mice. Collectively, our findings highlight novel mechanisms underlying HFD-related memory impairment and provide evidence of the potential therapeutic effect of exo-NSC against metabolic disease-related cognitive decline.

## 1. Introduction

The central nervous system undergoes structural and functional changes throughout adulthood in response to physiological stimuli and environmental conditions. Neuroplasticity involves proliferation and differentiation of adult neural stem cells as well as changes in the morphology and activity of differentiated neurons [1]. Environmental stimuli influence the physiology of brain cells by regulating the expression of large numbers of specific gene sets via epigenetic modifications [2]. Overnutrition alters normal cell signalling in the brain, potentially interfering with both synaptic function and adult neurogenesis, thereby leading to impairment of cognitive functions [3]. Accordingly, epidemiological evidence indicates that metabolic disorders, such as insulin resistance and type 2 diabetes, accelerate brain ageing and increase the risk of neurodegenerative diseases [4,5]. However, the molecular mechanisms underlying the long-term effects of nutrient excess on synaptic plasticity and memory are still partially unknown.

BDNF plays a pivotal role inside the brain, being upregulated in response to neuronal activity and enhancing both synaptic and structural plasticity via activation of the cAMP-PKA-CREB pathway [6,7]. The CREB-BDNF pathway has been clearly demonstrated to be fundamental to sustain neuron survival, synaptic plasticity and memory [8,9]. CREB has also been reported to work as a metabolic sensor by adapting the transcriptional activity in brain cells according to the nutrient availability [10,11]. In the last decades, multiple approaches targeting CREB and synaptic-plasticity-related pathways have been theorized and tested to counteract brain ageing and enhance cognitive functions [12,13]. More recently, several studies have shown that administration of stem cells-derived exosomes mitigates neural damage and stimulates functional recovery in animal models of brain disorders [14,15,16,17]. Exosomes are extracellular vesicles of endosomal origin that play a role in cell-to-cell communication, carrying information including hormones, peptides and microRNA [18]. Interestingly, BDNF may be secreted outside the cells as free protein or via exosomal vesicles [19], which raises the possibility of developing a BDNF-based therapeutic strategy exploiting the use of exosomes.

However, there is no evidence about the effects of stem cell-derived exosomal cargo on neuronal gene expression and brain function in a well-established mouse model of metabolic disease-dependent cognitive impairment, with mice fed a high fat diet (HFD) [20]. Here, we demonstrated that a HFD inhibited the CREB-BDNF pathway and reduced the expression of a set of synaptic plasticity-related CREB target genes in the hippocampus of mice. Moreover, intranasal administration of neural stem cell (NSC)-derived exosomes (exo-NSC) restored both CREB activity and TrkB signalling in the hippocampi of HFD mice, thus counteracting the HFD-dependent alteration of gene expression and memory deficits.

## 2. Results

### 2.1. HFD Impairs the Expression of Synaptic Plasticity-Related Genes

**Figure 1:**
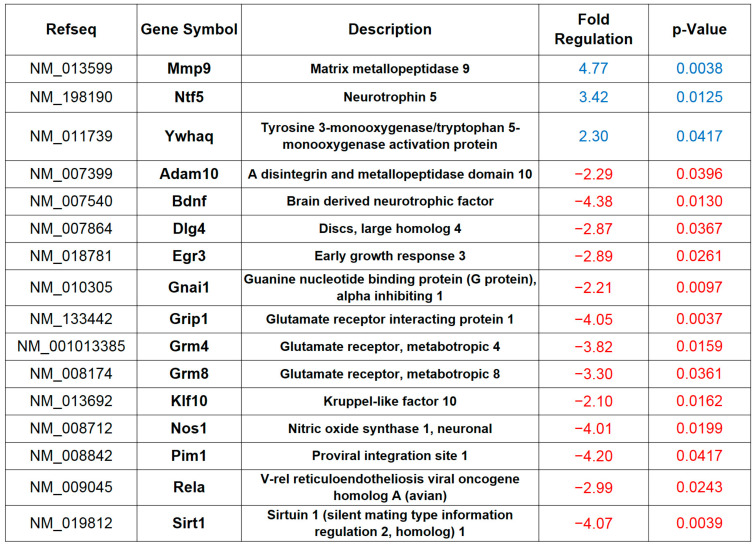
High fat diet (HFD) alters the expression of synaptic plasticity-related genes. Up-down fold expression changes of synaptic plasticity-related genes significantly altered in the hippocampus of mice fed with HFD for six weeks (*n* = 4 mice per experimental group). Real-time (RT)-PCR was performed in triplicate. The table shows genes with fold change ≥2 and *p* value < 0.05. The full list of genes and fold expression changes is shown in Supplementary Table S1.

Nutrient-dependent signals may influence cell physiology by both altering post-translational modifications of proteins and changing gene expression [21]. We recently reported that HFD affects hippocampal synaptic plasticity and memory by inhibiting the early phase of long-term potentiation (LTP) via AMPA receptor GluA1 hyper-palmitoylation [22]. However, the protein synthesis-dependent late-LTP and long-term memory require selective activation or repression of specific genes [23]. To gain insight into the mechanisms underlying the memory deficits due to the hypercaloric diet, we analysed the expression of a large number of plasticity-related genes in hippocampal extracts of mice fed a HFD for six weeks. A real-time PCR (RT-PCR) array revealed up- and downregulation of several genes in the hippocampus of overfed mice, including matrix metallopeptidase 9 (Mmmp9, +377%), neurotrophin 5 (Ntf5, +242%), Bdnf (−77%), discs large homolog 4 (Dlg4, −65%), early growth response 3 (Egr3, −65%), glutamate receptor interacting protein 1 (Grip1, −75%), glutamate metabotropic receptor 4 and 8 (Grm4 and Grm8, −74% and −70%, respectively), neuronal nitric oxide synthase (nNos, −75%), proviral integration site 1 (Pim1, −76%), v-rel reticuloendotheliosis viral oncogene homolog A (RelA, −66%) and sirtuin 1 (Sirt1, −75%) (*n* = 4 mice; Figure 1 and Supplementary Table S1). Collectively, the data from an unbiased analysis of gene expression indicated that HFD interfered with different molecular cascades potentially involved in the regulation of synaptic plasticity and memory.

### 2.2. HFD Downregulates CREB-BDNF-TrkB Signalling in the Hippocampus

**Figure 2:**
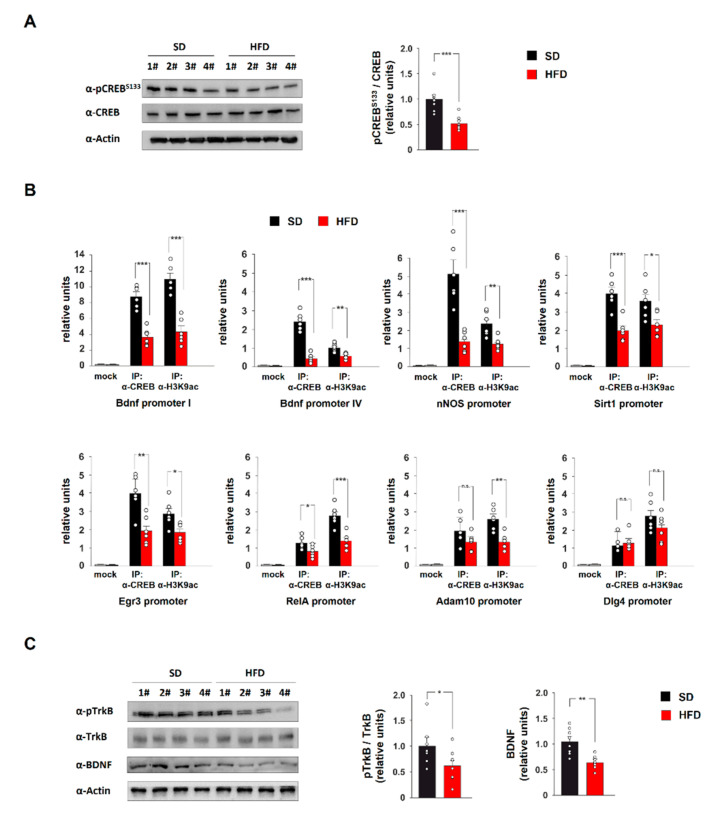
CREB-BDNF-TrkB signalling is inhibited in the hippocampus of HFD-fed mice. (**A**) Immunoblot analysis and bar graphs showing pCREB^S133^ in the hippocampus of mice fed with standard diet (SD) or HFD (*n* = 8 mice per group; statistics using unpaired Student’s *t* test). (**B**) ChIP assays of both CREB and histone 3 lysine 9 acetylation (H3K9ac) on the promoters of Bdnf, nNOS, Sirt1, Egr3, RelA, Adam10 and Dlg4 genes in the hippocampus of SD and HFD mice (*n* = 6 mice per group; statistics using unpaired Student’s *t* test). Mock indicates the binding of non-specific IgG. Real-time analysis was performed in triplicate. (**C**) Immunoblot analysis and bar graphs showing the levels of BDNF and pTrkB^T816^ in the hippocampus of SD and HFD mice (*n* = 8 mice; statistics using unpaired Student’s *t* test). Data are expressed as mean ±SEM. * *p* < 0.05; ** *p* < 0.01; *** *p* < 0.001; n.s., not significant.

Bioinformatic analysis of the RT-PCR array results identified a panel of potential CREB target genes among those differentially expressed in the hippocampus of HFD-fed mice (i.e., Bdnf, nNOS, Sirt1, Egr3, Adam10, Dlg4 and RelA). The activity of transcriptional factor CREB has been shown to be regulated by nutrient availability in neuronal cells [11,24]. Therefore, we analysed the activating phosphorylation of CREB at serine 133 (pCREB^Ser133^) in the hippocampus of HFD animals. The immunoblot analysis showed reduced levels of pCREB^Ser133^ in HFD mice compared to controls (−47.7%, *p* = 2.6 × 10^−4^; *n* = 8 mice; Figure 2A).

Since phosphorylation regulates the recruitment of CREB on the promoters of target genes, we analysed the binding of this transcription factor on the regulatory sequences of Bdnf, nNOS, Sirt1, Egr3, Adam10, Dlg4 and RelA. Chromatin immunoprecipitation (ChIP) analysis performed on the hippocampi of HFD mice revealed a decrease of CREB protein on the promoters of Bdnf, nNOS, Sirt1, Egr3 and RelA (Bdnf promoter I: −58.3%, *p* = 2.9 × 10^−5^; Bdnf promoter IV: −81.3%, *p* = 3.2 × 10^−6^; nNOS promoter: −73.1%, *p* = 5.3 × 10^−4^; Sirt1 promoter: −51.5%, *p* = 4.2 × 10^−4^; Egr3 promoter: −51.3%, *p* = 0.0027; RelA promoter: −38.1%, *p* = 0.024; *n* = 6 mice; Figure 2B), whereas no significant changes were observed on regulatory sequences of Adam10 and Dlg4 genes. Accordingly, the acetylation of lysine 9 on histone 3 (H3K9ac), an epigenetic marker of transcriptional activity, was significantly reduced on the same loci (Bdnf promoter I: −60.7%, *p* = 3.8 × 10^−5^; Bdnf promoter IV: −43.6%, *p* = 0.0024; nNOS promoter: −47.1%, *p* = 0.004; Sirt1 promoter: −35.3%, *p* = 0.022; Egr3 promoter: −36.2%, *p* = 0.016; RelA promoter: −49.3%, *p* = 8.1 × 10^−4^; *n* = 6 mice; Figure 2B). Bdnf represents a key CREB target gene but it also stimulates the transcription factor via activation of TrkB [25]. To investigate the molecular effects of CREB inactivation in hippocampi of HFD mice, we explored both the BDNF protein levels and the activating phosphorylation of TrkB at tyrosine 816 (pTrkB^Tyr816^). We found lower levels of both BDNF and pTrkB^Tyr816^ in HFD-fed animals compared to controls (−38.7%, *p* = 0.001 and −37.7%, *p* = 0.04, respectively; *n* = 8 mice; Figure 2C). Our data demonstrated that HFD inhibited CREB activity and reduced BDNF signalling leading to lower CREB-mediated transcription of key neuronal genes in the hippocampus.

### 2.3. NSC-Derived Exosomes Restore CREB Transcriptional Activity in HFD Mice

Synaptic plasticity in neurons is orchestrated by activation of multiple transcription factors [26]. Adult NSCs represent a source of neurotrophic factors released via exosomes potentially regulating the transcription in mature neurons [27,28,29]. Therefore, we tested the ability of exosomal vesicles derived from NSC (exo-NSC) to counteract HFD-dependent molecular changes.

**Figure 3:**
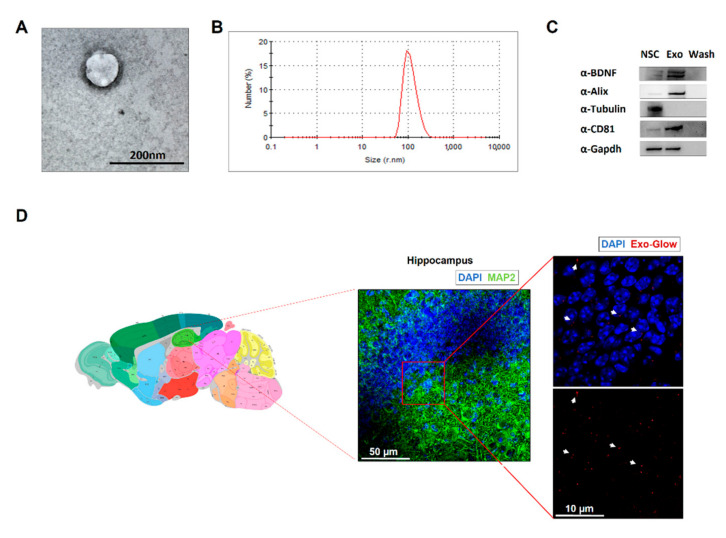
Neural stem cell (NSC)-derived exosomes characterization. (**A**) Transmission electron microscopy image of exosome isolated from NSC medium (exo-NSC). (**B**) Dynamic light scattering spectrum showing the size distribution curve of exo-NSC. (**C**) Representative images of immunoblots analysis of protein lysates extracted from exo-NSC, NSC or washing solution after exosome isolation (See Materials and Methods). CD81 and Alix are exosomal markers whereas Tubulin is used as negative control. (**D**) Confocal images of hippocampus of mice intranasally treated with exo-NSC labelled with fluorescent dye (Exo-Glow, red) and immunofluorescently stained for MAP2 (green). Arrows show exosomal vesicles. Image credit: Allen Institute (http://mouse.brain-map.org/static/atlas) [30].

**Figure 4:**
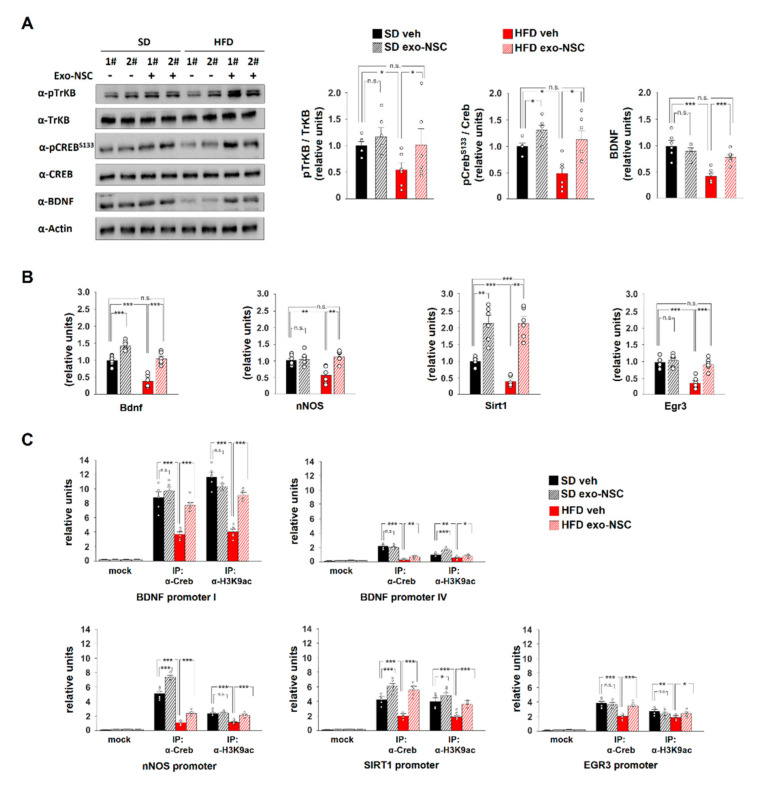
exo-NSCs restore CREB transcriptional activity in the hippocampus. (**A**) Immunoblot analysis and bar graphs showing the levels of BDNF, pTrkB^T816^ and pCreb^S133^ in mice intranasally treated with saline (vehicle) or exo-NSC and fed for 6 weeks with either SD or HFD (SD_veh_, SD_exo-NSC_, HFD_veh_, HFD_exo-NSC_, respectively; *n* = 6 mice per experimental group; statistics using two-way ANOVA and Bonferroni post hoc). (**B**) mRNA expression of Bdnf, nNos, Sirt1 and Egr3 in the hippocampus of SD_veh_, SD_exo-NSC_, HFD_veh_ and HFD_exo-NSC_ mice. Real-time analysis was performed in triplicate. The experiment was repeated six times using independent RNA samples (*n* = 6 mice per experimental group; statistics using two-way ANOVA and Bonferroni post hoc). (**C**) ChIP assays of both CREB and H3K9ac on the promoters of Bdnf, nNos, Sirt1 and Egr3 genes in the hippocampus of SD_veh_, SD_exo-NSC_, HFD_veh_ and HFD_exo-NSC_ mice. Real-time analysis was performed in triplicate (*n* = 6 mice per experimental group; statistics using two-way ANOVA and Bonferroni post hoc). Data are expressed as mean ±SEM. * *p* < 0.05; ** *p* < 0.01; *** *p* < 0.001; n.s., not significant.

First, we characterized exosomes purified from NSC culture media using transmission electron microscopy, scanning electron microscopy and dynamic light scattering. The analysis of physical properties of extracellular vesicles showed a typical cup shape and a size distribution with an average peak at 100 nm (Figure 3A,B and Supplementary Figure S1A). Moreover, immunoblot analysis of exosome cargo detected exosome-specific markers such as CD81 and Alix along with large amounts of BDNF in protein lysates extracted from exo-NSC (Figure 3C). Then, we investigated the effects of chronic intranasal administration of exo-NSC on CREB-BDNF-TrkB signalling in the hippocampus of HFD mice. Mice intranasally treated with exo-NSC showed the localization of vesicles inside the hippocampus (Figure 3D). The levels of pCREB^Ser133^ were increased in the hippocampus of both SD and HFD mice after treatment with exo-NSC (F_3.28_ = 11.23, SD_veh_ vs. SD_exo-NSC_ *p* = 0.014, SD_veh_ vs. HFD_veh_ *p* = 2.8 × 10^−3^, HFD_veh_ vs. HFD_exo-NSC_ *p* = 0.014, SD_veh_ vs. HFD_exo-NSC_ *p* = 0.531; *n* = 6; Figure 4A). Importantly, exo-NSC administration did not per se affect calorie intake in animals (Supplementary Figure S2A). The levels of BDNF and pTrkB^Tyr816^ were also completely restored in HFD mice upon administration of exo-NSC (F_3.28_ = 16.76 for BDNF, SD_veh_ vs. HFD_veh_ *p* = 3.4 × 10^−4^, HFD_veh_ vs. HFD_exo-NSC_ *p* = 9.3 × 10^−4^, SD_veh_ vs. HFD_exo-NSC_ *p* = 0.062; F_3.28_ = 6.61 for pTrkB^Tyr816^, SD_veh_ vs. HFD_veh_ *p* = 0.026, HFD_veh_ vs. HFD_exo-NSC_ *p* = 0.032, SD_veh_ vs. HFD_exo-NSC_ *p* = 0.689; *n* = 6; Figure 4A).

Subsequently, we evaluated the transcription of CREB target genes that we found downregulated in the hippocampus of HFD mice. Administration of exo-NSC increased in HFD mice the expression of Bdnf, nNOS and Egr3 to levels comparable with controls (F_3.28_ = 34.57 for Bdnf, SD_veh_ vs. HFD_veh_ *p* = 7.4 × 10^−5^, HFD_veh_ vs. HFD_exo-NSC_ *p* = 7.9 × 10^−5^, SD_veh_ vs. HFD_exo-NSC_ *p* = 0.581; F_3.28_ = 9.48 for nNOS, SD_veh_ vs. HFD_veh_ *p* = 5.1 × 10^−3^, HFD_veh_ vs. HFD_exo-NSC_ *p* = 1.4 × 10^−3^, SD_veh_ vs. HFD_exo-NSC_ *p* = 0.302; F_3.28_ = 20.5 for Egr3, SD_veh_ vs. HFD_veh_ *p* = 6.4 × 10^−5^, HFD_veh_ vs. HFD_exo-NSC_ *p* = 2.3 × 10^−4^, SD_veh_ vs. HFD_exo-NSC_ *p* = 0.565; *n* = 6; Figure 4B). Treatment with exo-NSC stimulated the expression of Bdnf and Sirt1 also in SD mice (F_3.28_ = 34.57 for Bdnf, SD_veh_ vs. SD_exo-NSC_ *p* = 2.7 × 10^−4^; F_3.28_ = 26. 71 for Sirt1, SD_veh_ vs. SD_exo-NSC_ *p* = 0.0021, SD_veh_ vs. HFD_veh_ *p* = 2 × 10^−5^, HFD_veh_ vs. HFD_exo-NSC_ *p* = 0.0001, SD_veh_ vs. HFD_exo-NSC_ *p* = 9.5 × 10^−4^; *n* = 6; Figure 4B). Conversely, no significant changes were observed in the expression of RelA, Adam10 and Dlg4 after exo-NSC administration (Figure S2B). Moreover, intranasal administration of exo-NSC counteracted the HFD-dependent downregulation of nNos, Bdnf and Sirt1 in the neocortex (F_3.28_ = 30.17 for Bdnf, SD_veh_ vs. HFD_veh_ *p* = 1.3 × 10^−4^, HFD_veh_ vs. HFD_exo-NSC_ *p* = 0.016, SD_veh_ vs. HFD_exo-NSC_ *p* = 0.148; F_3.28_ = 42.35 for nNOS, SD_veh_ vs. HFD_veh_ *p* = 6.3 × 10^−5^, HFD_veh_ vs. HFD_exo-NSC_ *p* = 2.97 × 10^−4^, SD_veh_ vs. HFD_exo-NSC_ *p* = 0.105; F_3.28_ = 34.34 for Sirt1, SD_veh_ vs. HFD_veh_ *p* = 4.1 × 10^−4^, HFD_veh_ vs. HFD_exo-NSC_ *p* = 0.005, SD_veh_ vs. HFD_exo-NSC_ *p* = 0.428; *n* = 6; Figure S2C).

To further investigate the effect of exo-NSC on CREB transcriptional activity, we analysed the binding of the transcription factor on the promoters of genes modulated by exo-NSC. ChIP experiments showed that exo-NSC treatment increased the recruitment of CREB on the promoters of Bdnf, nNOS, Sirt1 and Egr3 in HFD mice (F_3.28_ = 27.04 for Bdnf promoter I, HFD_veh_ vs. HFD_exo-NSC_ *p* = 2.1 × 10^−5^; F_3.28_ = 84.53 for Bdnf promoter IV, HFD_veh_ vs. HFD_exo-NSC_ *p* = 0.0038; F_3.28_ = 214.95 for nNOS, HFD_veh_ vs. HFD_exo-NSC_ *p* = 2.4 × 10^−4^; F_3.28_ = 34.7 for Sirt1, HFD_veh_ vs. HFD_exo-NSC_ *p* = 2.1 × 10^−5^; F_3.28_ = 18.86 for Egr3, HFD_veh_ vs. HFD_exo-NSC_ *p* = 1.4 × 10^−5^; *n* = 6; Figure 4C). Accordingly, H3K9ac was enhanced on the same regulatory sequences after exo-NSC administration (F_3.28_ = 68.25 for Bdnf promoter I, HFD_veh_ vs. HFD_exo-NSC_ *p* = 4.9 × 10^−7^; F_3.28_ = 23.35 for Bdnf promoter IV, HFD_veh_ vs. HFD_exo-NSC_ *p* = 0.038; F_3.28_ = 19.71 for nNOS, HFD_veh_ vs. HFD_exo-NSC_ *p* = 1.2 × 10^−4^; F_3.28_ = 22.66 for Sirt1, HFD_veh_ vs. HFD_exo-NSC_ *p* = 1.3 × 10^−4^; F_3.28_ = 5.17 for Egr3, HFD_veh_ vs. HFD_exo-NSC_ *p* = 0.037; *n* = 6; Figure 4C). Collectively, our findings demonstrated that exo-NSC stimulated CREB-BDNF-TrkB signalling in the hippocampus of HFD mice leading to enhancement of CREB recruitment and transcriptional activity on the promoters of synaptic plasticity-related genes.

### 2.4. NSC-Derived Exosomes Counteract HFD-Dependent Memory Deficits

**Figure 5:**
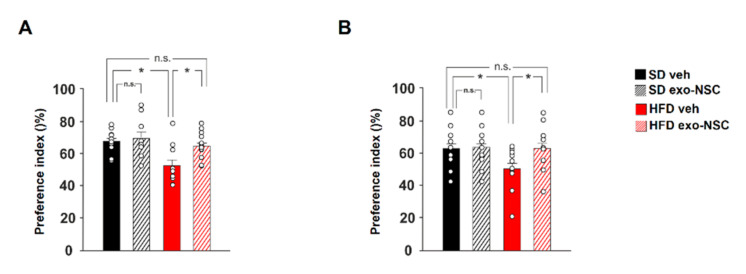
NSC-derived exosomes counteract HFD-related memory deficits. (**A**) Preference index for the novel object in the novel object recognition (NOR) paradigm in SD and HFD mice treated with vehicle or exo-NSC (*n* = 10 for SD vehicle, 9 SD exo-NSC, 10 for HFD vehicle, 15 HFD exo-NSC; statistics using two-way ANOVA and Bonferroni post hoc). (**B**) Preference index for the displaced object in the object place recognition (OPR) paradigm in SD and HFD mice treated with vehicle or exo-NSC (*n* = 10 for each group; statistics using two-way ANOVA and Bonferroni post hoc). Data are expressed as mean ±SEM. * *p* < 0.05; n.s., not significant.

Looking for a behavioural correlate of the molecular data reported above we tested whether exosomes secreted from NSCs could prevent the cognitive impairment induced by HFD. To this aim, we performed cognitive tasks such as novel object recognition (NOR) and object place recognition (OPR) tests in overfed mice after intranasal administration of exo-NSC. In the NOR test, HFD mice showed a significantly higher preference toward the novel object upon treatment with exo-NSC (F_3.008_ = 3.92, SD_veh_ vs. HFD_veh_ *p* = 0.011, HFD_veh_ vs. HFD_exo-NSC_ *p* = 0.034; *n* = 9; Figure 5A). Moreover, exo-NSC treatment completely abolished the detrimental effects of HFD on spatial memory, evaluated using the OPR test (F_3.008_ = 3.44, SD_veh_ vs. HFD_veh_ *p* = 0.036, HFD_veh_ vs. HFD_exo-NSC_ *p* = 0.013; *n* = 9; Figure 5B). Collectively, our findings demonstrated that exosomal cargo derived from NSCs counteracted the HFD-dependent downregulation of CREB transcriptional activity and rescued the overnutrition-related memory deficits.

## 3. Discussion

Epidemiological and molecular evidence demonstrates that overnutrition and metabolic diseases negatively impact on hippocampal synaptic plasticity leading to alteration of cognitive functions [31]. Several molecular mechanisms have been proposed to be involved in nutrient excess-related cognitive impairment, including mitochondrial dysfunction, activation of pro-inflammatory cytokines, development of brain insulin resistance and alteration of neurotrophin signalling [22,32,33,34]. However, how nutrient overload impinges on neuronal gene expression causing long-lasting effects on synaptic plasticity and memory has not been understood yet. Here, we investigated the expression of a large number of synaptic plasticity-related genes in the hippocampus of a well-established animal model of metabolic disorder, HFD-fed mice. The unbiased analysis revealed reduced expression of several genes responsive to the cellular energy state (e.g., Sirt1, Bdnf, and nNOS) and regulating the synaptic strength (e.g., Egr3, RelA and Dlg4) (Figure 1). More importantly, bioinformatic analysis of RT-PCR array data revealed a potential CREB-driven regulation of a cluster of downregulated genes. Accordingly, we found reduced levels of pCREB^Ser133^ in the hippocampus of HFD-fed mice (Figure 2A) and decrease of both binding of the transcription factor and acetylation of H3K9 (i.e., a marker of transcriptional activity) on the regulatory sequences of several memory-related genes (Figure 2B).

Furthermore, HFD also caused lower expression of the neurotrophic factor BDNF and inhibition of its molecular cascade (Figure 2C). Interestingly, activation of downstream effectors of the BDNF pathway cell autonomously increased both BDNF synthesis and release, leading to a feed-forward TrkB-mediated signalling loop that amplifies synaptic strength [35]. Accordingly, injection of function-blocking anti-BDNF antibody into the CA1 region of the hippocampus decreased CREB phosphorylation and impaired memory formation [36]. Our data support the role of CREB as a metabolic sensor in the brain. It has been demonstrated that calorie restriction induces CREB activation and enhances memory by inducing CREB-mediated gene expression [37]. Conversely, streptozotocin-induced hyperglycaemia inhibited CREB activation and impaired synaptic functions [11]. Our findings reveal that HFD impinges on CREB-BDNF-TrkB signalling causing alteration of synaptic plasticity-related gene expression.

A rising number of pre-clinical studies have proposed transplantation of stem cell-derived exosomes as a new therapeutic strategy against cognitive decline and neurodegeneration. For instance, the intranasal administration of exosomes derived from mesenchymal stem cells (MSCs) in a mouse model of Alzheimer’s disease (AD) rescued dendritic spine density by reducing the inflammation [38]. Accordingly, exosomes derived from adipose MSCs enhanced Aβ clearance in vitro [39]. NSC-derived exosomal cargo contains a plethora of bioactive molecules potentially contributing to regulation of transcriptional activity and synaptic activity in neurons, including BDNF [40]. Intranasal administration of exo-NSC rescued CREB activation and neurotrophin-associated molecular cascade in the hippocampus of HFD mice (Figure 4A). Increase of CREB phosphorylation and changes of gene expression have been also detected in primary cortical neurons upon treatment with oligodendrocyte-derived exosomes [41]. Moreover, MSC-derived exosomes have been demonstrated to carry miR-133b, which positively modulates ERK1/2, STAT3 and CREB activation [42]. In addition, in vivo administration of exosomes increased neural plasticity and functional recovery after stroke by transfer of microRNAs including miR-17-92 and miR-133b [43,44]. Exosomal cargo can stimulate in the target cells the expression of neurotrophic factors promoting neural activity such as NGF and S100b [45]. Exo-NSC administration also restored the levels of a large number of key genes downregulated by HFD in the hippocampus of mice and reverted the overnutrition-related memory impairment (Figure 4B and Figure 5A,B). Similarly, an enriched environment counteracted diabetes-induced cognitive impairment by inducing secretion of exosomal miR-146a from MSCs [46]. Interestingly, intranasal administration of exo-NSCs mimicked calorie restriction-related signals in the hippocampus without changing the calorie intake of mice (Figure S2). Indeed, NSC-derived vesicles induced the expression of nutrient deprivation-related molecules such as Sirt1 and Bdnf (Figure 4B). Accordingly, the calorie restriction mimetic resveratrol has been demonstrated to improve cognitive functions by reducing the expression of miRNA inhibiting the CREB-BDNF pathway [47].

Collectively, our paper provides novel evidence that exo-NSC counteracts HFD-induced memory impairment by modulating the CREB-dependent expression of synaptic plasticity-related genes. Extracellular vesicles represent a more promising tool than stem cell transplantation against age-related diseases due to the lower risk of tumorigenicity and side effects [48]. However, further studies are necessary to better characterize the composition of exosomal cargo derived from stem cells and to understand the therapeutic potential of each component for personalized medicine.

## 4. Materials and Methods

### 4.1. Ethics and Animal Use Statement

Male C57BL/6 mice (30–35 days-old), derived from the Animal Facility of Università Cattolica del Sacro Cuore, were used and randomly assigned to two feeding regimens: (i) standard diet (SD, control) and (ii) high fat diet (HFD). Different groups of mice were used for each experimental test. Mice were always housed in groups (3–5 animals per cage) and they were monitored daily. All animal procedures were reviewed and approved on 16 January 2017 by the Ethics Committee of Università Cattolica del Sacro Cuore and were fully compliant with Italian (Ministry of Health guidelines, Legislative Decree No. 116/1992) and European Union (Directive No. 86/609/EEC) legislations on animal research. The methods were carried out in strict accordance with the approved guidelines. The animals were housed under a 12-h light-dark cycle at room temperature (RT: 19–22 °C), fed with their respective diet and water ad libitum and body weight was monitored weekly.

### 4.2. Animals and Treatments

Mice from the same litter were randomly assigned to different experimental groups. Animals were fed with SD (18.5% proteins; 46% carbohydrates, namely 42% starch, 4% sucrose; 3% fats; 6.55% fat caloric content; cat. num. 4RF21) or HFD (23% proteins; 42% carbohydrates, namely 28% starch, 9% sucrose, 5% maltodextrin; 34% fats; 60% fat caloric content; cat. num. PF4051/D) for 6 weeks. The diets were from Mucedola (Guidonia Montecelio, RM, Italy). For in vivo administration of NSC-derived exosomes (exo-NSC), mice were intranasally treated with saline (vehicle) or exo-NSC (1.5 µg per nostril, 3 times per week) for the entire duration of the diet. Mice used for molecular analyses were immediately sacrificed at the end of the diet.

### 4.3. Culture of Neural Stem Cells

Postnatal hippocampal NSC culture were isolated according to previously published protocol [10]. Briefly, brains of newborn (0–1 day old) C57bl/6 mice were microdissected to obtain the hippocampal region upon sagittal sectioning. Tissues were finely minced and digested using accutase (in DPBS, 0.5 mM EDTA; Innovative Cell Tecnologies, Inc., San Diego, CA, USA) at 37 °C for 30 min. After centrifugation, cells were carefully dissociated by passaging in fire-polished Pasteur pipettes and resuspending in NeurobasalA medium, supplemented by 2% B27 (Gibco, Grand Island, NY, USA), Glutamax (0.5 mM; Invitrogen, Carlsbad, CA, USA), mouse fibroblast growth factor 2 (FGF2, 10 ng/mL; Invitrogen), epidermal growth factor (EGF, 10 ng/mL; Invitrogen, Carlsbad, CA, USA) and mouse platelet-derived growth factor bb (PDGFbb; 10 ng/mL; Invitrogen, Carlsbad, CA, USA). Cells were seeded onto a 25-cm^2^ T-flask and incubated at 37 °C in 5% CO_2_ atmosphere. During the first week NSCs began to form neurospheres in vitro. At 2-day intervals, the neurospheres were collected and passaged using a gently enzymatic and mechanical dissociation. After 1–2 passages of NSC expansion, NSC medium containing extracellular vesicles was separated via centrifugation (800× *g* 10 min). NSCs cultured in the medium described above, thereinafter referred to as “proliferation medium”, remained in an undifferentiated state and were proliferated.

### 4.4. Exosomes Isolation

Twenty-four hours after medium change, the media were collected and immediately frozen at −80°C until exosome isolation. exo-NSC were isolated from culture medium using an exoEasy Maxi Kit (Qiagen) and multistep centrifugations according to the manufacturer indications. The exosomes were quantified with the Bradford method.

### 4.5. Dynamic Light Scattering

Exosome analyses were performed using Zetasizer Nano ZS apparatus (Malvern Instruments Ltd., Worcestershire, UK). Data for each sample were collected on a continuous basis for 12 min in sets of four measurements for each sample [49].

### 4.6. Transmission Electron Microscopy

Morphological analysis of exosomes was performed using transmission electron microscopy. The isolated samples were fixed with formaldehyde and 2.5% glutaraldehyde in 0.1 mol/L sodium cacodylate buffer (pH 7.4) and then placed on Formvar-carbon-coated grids and air-dried for 10 min. After being rinsed with double-distilled water, the exosomes were postfixed in 1.5% osmium tetroxide in 0.1 mol/L cacodylate buffer (pH 7.3) and then were allowed to dry. Vesicles were observed with a Zeiss Libra 120 (Zeiss NTSGmbH, Oberkochen, Germany).

### 4.7. Scanning Electron Microscope

Exosomes were fixed on grids using a buffer with 4% of paraformaldehyde and 2% of glutaraldehyde. PBS buffer was added three times to wash the sample. After, grids were serially washed with 25%, 50%, 70%, 95%, 100% and 100% ethanol solutions on ice for 5 min. To make the surface conductive, a coating of gold-palladium alloy was applied (10 mA for 15 s) before imaging. High-resolution images were acquired with the following settings: accelerating voltages = 6 kV, working distance = 10 nm and magnification = 122.000×. Vesicles were observed with a Zeiss supra 25 (Zeiss NTSGmbH, Oberkochen, Germany).

### 4.8. Behavioural Experiments

Behavioural tests were carried out from 9 a.m. to 4 p.m. and data were analysed blind using an automated video tracking system (Any-Maze^™^). Recognition memory was evaluated using a novel object recognition (NOR) test. On the first day, animals were familiarized for 10 min with the test arena (45 × 45 cm). On the second day (training session), they were allowed to explore two identical objects placed symmetrically in the arena for 10 min. Mice that took less than 20 s as total exploration time or that explored one of two identical objects for more than 10% of the total exploration time during the training session were excluded from the test. On the third day (test session), a new object replaced one of the old objects. Animals were allowed to explore for 10 min and the preference index, calculated as the ratio between time spent exploring the novel object and time spent exploring both objects, was used to measure recognition memory. To exclude place preference in the test session, the position of the novel object was alternated on both sides of the box. All objects and the box were cleaned with 70% ethanol solution at the end of each test.

Spatial memory was analysed using an object place recognition (OPR) test. The animals were first habituated for 10 min to the testing arena. Different cues were placed on the walls of the testing arena in order to provide spatial points of reference. In the training phase, 24 h after the habituation phase, the animals were exposed to a couple of identical objects in two corners of the arena. After 10 min, the animals were removed from the testing arena and taken back to their home cage. Twenty-four hours after the training phase, one of the objects was moved to the opposite corner and the animals were brought back to the testing area for 10 min. As for the NOR test, the time spent exploring both objects was recorded and a preference index for the displaced object was calculated. Between each animal, the objects and the arena were cleaned with 70% ethanol solution and fresh bedding was added.

### 4.9. Western Blotting

Hippocampi and exosomes were homogenized in ice-cold lysis buffer (NaCl 150 mM, Tris-HCl 50 mM pH 7.4, EDTA 2 mM) containing 1% Triton X-100, 0.1% SDS, 1× protease inhibitor cocktail (Sigma-Aldrich, St. Louis, MO, USA), 1 mM sodium orthovanadate (Sigma-Aldrich, St. Louis, MO, USA) and 1 mM sodium fluoride (Sigma-Aldrich, St. Louis, MO, USA). The homogenized was sonicated for 10 s “on” and 20 s “off” 3 times using a Diagenode Bioruptor Standard Waterbath Sonicator, and then, the sample was spun down at 22,000× *g*, 4 °C. Supernatant was quantified for protein content (DC Protein Assay; Bio-Rad, Hercules, CA, USA). Equal amounts of protein were diluted in Laemmli buffer, boiled and resolved using SDS-PAGE as previously described [50]. The primary antibodies (available in Table S2) were incubated overnight and revealed with HRP-conjugated secondary antibodies (Cell Signaling Technology Inc., Danvers, MA, USA) and chemiluminescent substrates (Cyanagen, Bologna, BO, Italy). The band density was documented and quantified using UVItec Cambridge Alliance. Expression levels of the target protein were quantified by calculating the band intensity ratio of the target protein and actin (loading control) in each lane. Phosphorylation levels of the target protein were quantified by calculating the band intensity ratio of the phospho-target protein, target protein and actin (loading control) in each lane. In each bar graph, the mean value of controls was set to 1 and the expression or phosphorylation levels of the target protein were shown as fold changes compared to the control (relative units). Images shown were cropped for presentation with no manipulations.

### 4.10. RNA Analyses

Hippocampi and neocortices were isolated under optic microscope and homogenized in TRIzol (Invitrogen, Carlsbad, CA, USA). RNA was extracted and purified using an RNeasy Mini Kit (Qiagen, Hilden, Germany) according to the manufacturer’s instructions. cDNA was synthetized using an RT2 First Strand Kit (Qiagen, Hilden, Germany). For PCR array experiments, an RT2 Profiler Custom PCR Array (PAMM-126Z) was used to simultaneously examine the mRNA levels of 89 genes, including 5 housekeeping genes in 96-well plates according to the protocol of the manufacturer (Qiagen, Hilden, Germany). cDNA of all samples was analysed in triplicate, and data were normalized for actin levels using the ΔΔCt method. All results are shown in Table S1. Bioinformatic analysis of transcription factors regulating the modified genes was performed using RT^2^ Profiler Data Analysis Software (Qiagen, Hilden, Germany).

Quantitative real-time PCR (qRT-PCR) amplifications were performed using SYBR GREEN qPCR Master Mix (Fisher Molecular Biology, Roma, RM, Italy) on an AB7500 instrument (Life Technologies, Carlsbad, CA, USA) according to the manufacturer’s instructions. The thermal cycling profile featured a pre-incubation step of 94 °C for 10 min, followed by 40 cycles of denaturation (94 °C, 15 s), annealing (55 °C, 30 s) and elongation (72 °C, 20 s). Melting curves were subsequently generated (94 °C for 15 s, 50 °C for 30 s, slow heating to 94 °C in increments of 0.5 °C). Melting-curve analyses confirmed that only single products had been amplified. The primer sequences are shown in Table S3. All data were normalized by reference to the amplification levels of the actin; a reference dye was included in the SYBR master mix. RNA of all samples was analysed in triplicate. The thresholds calculated using the software were used to calculate specific mRNA expression levels using the cycle-at-threshold (Ct) method, and all results are expressed as fold changes (compared to control) for each transcript, employing the 2^−ΔΔCt^ approach.

### 4.11. Chromatin Immunoprecipitation

Chromatin immunoprecipitation (ChIP) assays were performed as previously described [34]. Hippocampi were homogenized in 200 μL lysis buffer containing 1% SDS, 50 mM Tris-HCl pH 8.0, and 10 mM EDTA and sonicated on ice with six 10-s pulses with a 20-s interpulse interval. Sample debris was removed via centrifugation, and supernatants were precleared with protein-G Sepharose 4B beads (Sigma-Aldrich, St. Louis, MO, USA) for 1 h at 4 °C. 2 μg of anti-CREB, anti acetil H3K9 or control IgG were added overnight at 4 °C. Immune complexes were collected via incubation with protein-G Sepharose 4B beads for 2 h at 4 °C. After seven sequential washes, immune complexes were eluted from beads by vortexing in elution buffer (1% SDS and NaHCO_3_ 0.1 M; pH 8.0). NaCl was added (final concentration 0.33 M), and cross-linking was reversed via incubation overnight at 65 °C. DNA fragments were purified using a PCR DNA fragments purification kit (Geneaid Biotech Ltd., New Taipei City, Taiwan). The primer sequences are shown in Table S3.

Bioinformatic analysis to identify putative cAMP response element (CRE) regions was performed online (http://natural.salk.edu/CREB/) using the CREB Target Gene Database [51]. PCR conditions and cycle numbers were determined empirically and each PCR reaction was performed in triplicate. Data are expressed as percentage of input calculated by the “adjusted input value” method according to the manufacturer’s instructions (ThermoFisher Scientific ChIP Analysis, Carlsbad, CA, USA). To calculate the adjusted input the Ct value of the input was subtracted by 6.644 (i.e., log2 of 100). Next, the percent input of samples was calculated using the formula 100*2^(Adjusted input—Ct(ChIP). The percent input of IgG samples was calculated using the formula 100*2^(Adjusted input—Ct(IgG).

### 4.12. Exosome Labelling and Immunofluorescence

To track the EVs in the brain, the isolated EVs were labelled with the red fluorescent membrane dye kit ExoGlowTM (ExoGlowTM Membrane EV Labeling Kit, System Biosciences, Palo Alto, CA, USA), following the manufacturer’s instructions. Briefly, exosomes were incubated with a mixture of reaction buffer and labelling dye for 30 min at RT. Labelled exosomes were then separated from unbound fluorescent dye through PD-Spintrap G-25 (GE Healthcare, Chicago, IL, USA) following manufacturer’s instructions.

For in vivo localization of exo-NSC, mice were intranasally treated 3 times (8 µg per nostril) at half an hour from each other. Six hours after the last administration the animals were deeply anesthetized with ketamine and xylazine and were transcardially perfused with PBS (0.1 M, pH 7.4) followed by 4% paraformaldehyde (PFA). Brains were collected, post-fixed overnight at 4 °C in PFA, and then transferred to a solution of 30% sucrose in 0.1 M PBS. Sagittal sections (40 μm) were then obtained using a cryostat (SLEE, Mainz, Germany) and subsequently stored at 4 °C in PBS until use.

Immunohistochemistry was performed as previously described [10]. After permeabilization and blocking (1-h incubation with 0.3% Triton X-100 (Sigma, St. Louis, MO, USA) in PBS and 5% Normal Goat Serum), tissues were incubated overnight at 4 °C with MAP-2 antibody (1:400, Sigma, St. Louis, MO, USA). The next day, tissues were incubated for 90 min at RT with the secondary antibody: Alexa Fluor 488 donkey anti-mouse (1:500; Invitrogen, Carlsbad, CA, USA). Finally, nuclei were counterstained with 4′,6-diamidino-2-phenylindole (DAPI, 0.5 μg/mL for 10 min; Invitrogen, Carlsbad, CA, USA), and slices were coverslipped with ProLong Gold anti-fade reagent (Invitrogen, Carlsbad, CA, USA). Images (1024 × 1024 pixels) were acquired at 60× magnification with a Nikon A1 MP confocal system (Tokyo, Japan) and an oil-immersion objective (N.A. 1.2). For some images, additional 3× magnification was applied.

### 4.13. Statistical Analysis

Sample sizes were chosen with adequate power (0.8) according to results of prior pilot data sets or studies, including our own, which used similar methods or paradigms. Sample estimation and statistical analyses were performed using SigmaPlot 14 software. Data were first tested for equal variance and normality (Shapiro-Wilk test) and the appropriate statistical tests were chosen. The statistical tests used (i.e., Student’s *t*-test, two-way ANOVA) are indicated in the main text and in the corresponding figure legends for each experiment. N numbers are reported in the figure legends. Degrees of freedom are n-1 for each condition in both the unpaired *t*-test and ANOVA tests. Post-hoc multiple comparisons were performed with Bonferroni correction. All statistical tests were two-tailed and the level of significance was set at 0.05. Results are shown as mean ±SEM.

## Supplementary Materials

The following are available online at https://www.mdpi.com/1422-0067/21/23/8994/s1.
