# Supplementary material for: Neural Stem Cell-Derived Exosomes Revert HFD-Dependent Memory Impairment via CREB-BDNF Signalling
Source: Int J Mol Sci. 2020 Nov 26;21(23):8994. doi: 10.3390/ijms21238994 (PMC7729830; doi:10.3390/ijms21238994)
Supplement: Supplementary file 1 [file ijms-21-08994-s001.pdf]

International Journal of
Molecular Sciences

MDPI

Article

Neural stem cell-derived exosomes revert HFD-dependent memory impairment via BDNF-CREB signalling

Matteo Spinelli [1], Francesca Natale [1,2], Marco Rinaudo [1], Lucia Leone [1,2], Daniele Mezzogori [1], Salvatore Fusco [1,2*], and Claudio Grassi [1,2]

[1] *Department of Neuroscience, Università Cattolica del Sacro Cuore, 00168 Rome, Italy*
[2] *Fondazione Policlinico Universitario A. Gemelli IRCCS, 00168 Rome, Italy*
* Correspondence: salvatore.fusco@unicatt,it

A

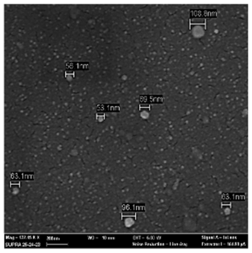

Figure S1. exo-NSC characterization. (A) Scanning electron microscope image for morphology and size characterization of exosome isolated from NSC medium (exo-NSC). The size of exo-NSC typically ranges from 30nm to 120 nm.

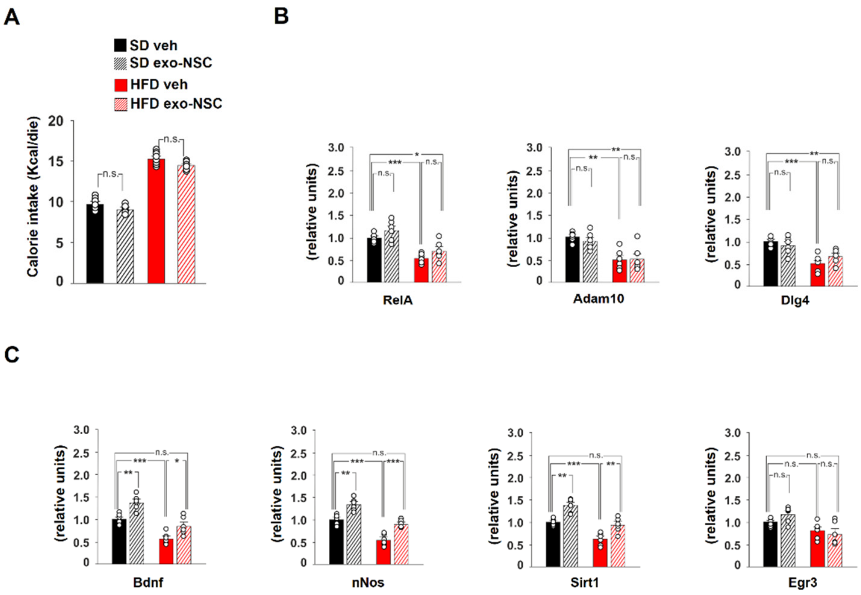

Figure S2. Effects of exo-NSC on gene expression. (A) Calorie intake (Kcal/die) (n = 8 mice per experimental group; statistics by two-way ANOVA and Bonferroni post hoc). (B) mRNA expression of RelA, Adam10, Dlg4 in the hippocampus of SD_{veh}, $SD_{exo\text{-}NSC}$, HFD_{veh} and $HFD_{exo\text{-}NSC}$ mice. Real Time analysis was performed in triplicate (n = 6 mice per experimental group; statistics by two-way ANOVA and Bonferroni post hoc). (C) mRNA expression of Bdnf, nNos, Sirt1 and Egr3 in the neocortex of SD_{veh}, $SD_{exo\text{-}NSC}$, HFD_{veh} and $HFD_{exo\text{-}NSC}$ mice. Real Time analysis was performed in triplicate (n = 6 mice per experimental group; statistics by two-way ANOVA and Bonferroni post hoc). Data are expressed as mean ± SEM. *$p < 0.05$; **$p < 0.01$; ***$p < 0.001$; n.s. not significant.

Supplementary Table 1. GENE REGULATION

Table S1. Fold changes of 84 genes analyzed in the hippocampus of HFD mice.

Refseq	Symbol	Description	HFD fold change
NM_007399	Adam10	A disintegrin and metallopeptidase domain 10	0.44
NM_009622	Adcy1	Adenylate cyclase 1	0.89
NM_009623	Adcy8	Adenylate cyclase 8	1.14
NM_009652	Akt1	Thymoma viral proto-oncogene 1	0.61
NM_018790	Arc	Activity regulated cytoskeletal-associated protein	1.95
NM_007540	Bdnf	Brain derived neurotrophic factor	0.23
NM_177407	Camk2a	Calcium/calmodulin-dependent protein kinase II alpha	1.11
NM_178597	Camk2g	Calcium/calmodulin-dependent protein kinase II gamma	1.24
NM_007664	Cdh2	Cadherin 2	1.04
NM_009883	Cebpb	CCAAT/enhancer binding protein (C/EBP), beta	0.70
NM_007679	Cebpd	CCAAT/enhancer binding protein (C/EBP), delta	1.45
NM_007726	Cnr1	Cannabinoid receptor 1 (brain)	0.85
NM_133828	Creb1	CAMP responsive element binding protein 1	0.60
NM_013498	Crem	CAMP responsive element modulator	1.61
NM_007864	Dlg4	Discs, large homolog 4 (Drosophila)	0.35
NM_007913	Egr1	Early growth response 1	0.73
NM_010118	Egr2	Early growth response 2	1.43
NM_018781	Egr3	Early growth response 3	0.35
NM_020596	Egr4	Early growth response 4	0.80
NM_010142	Ephb2	Eph receptor B2	1.27
NM_010234	Fos	FBJ osteosarcoma oncogene	1.39
NM_176942	Gabra5	Gamma-aminobutyric acid (GABA) A receptor, subunit alpha 5	1.11
NM_010305	Gnai1	Guanine nucleotide binding protein (G protein), alpha inhibiting 1	0.45
NM_008165	Gria1	Glutamate receptor, ionotropic, AMPA1 (alpha 1)	0.75
NM_013540	Gria2	Glutamate receptor, ionotropic, AMPA2 (alpha 2)	0.61
NM_016886	Gria3	Glutamate receptor, ionotropic, AMPA3 (alpha 3)	0.74
NM_019691	Gria4	Glutamate receptor, ionotropic, AMPA4 (alpha 4)	1.30
NM_008169	Grin1	Glutamate receptor, ionotropic, NMDA1 (zeta 1)	1.39
NM_008170	Grin2a	Glutamate receptor, ionotropic, NMDA2A (epsilon 1)	1.73
NM_008171	Grin2b	Glutamate receptor, ionotropic, NMDA2B (epsilon 2)	0.87

NM_010350	Grin2c	Glutamate receptor, ionotropic, NMDA2C (epsilon 3)	2.74
NM_008172	Grin2d	Glutamate receptor, ionotropic, NMDA2D (epsilon 4)	1.45
NM_133442	Grip1	Glutamate receptor interacting protein 1	0.25
NM_016976	Grm1	Glutamate receptor, metabotropic 1	1.18
NM_001160353	Grm2	Glutamate receptor, metabotropic 2	0.92
NM_181850	Grm3	Glutamate receptor, metabotropic 3	0.77
NM_001013385	Grm4	Glutamate receptor, metabotropic 4	0.26
NM_001081414	Grm5	Glutamate receptor, metabotropic 5	1.06
NM_177328	Grm7	Glutamate receptor, metabotropic 7	1.04
NM_008174	Grm8	Glutamate receptor, metabotropic 8	0.30
NM_152134	Homer1	Homer homolog 1 (Drosophila)	0.94
NM_010512	Igf1	Insulin-like growth factor 1	1.06
NM_008380	Inhba	Inhibin beta-A	1.09
NM_010591	Jun	Jun oncogene	1.32
NM_008416	Junb	Jun-B oncogene	0.52
NM_010623	Kif17	Kinesin family member 17	1.54
NM_013692	Klf10	Kruppel-like factor 10	0.48
NM_011949	Mapk1	Mitogen-activated protein kinase 1	1.16
NM_013599	Mmp9	Matrix metallopeptidase 9	4.77
NM_010875	Ncam1	Neural cell adhesion molecule 1	1.15
NM_008689	Nfkb1	Nuclear factor of kappa light polypeptide gene enhancer in B-cells 1, p105	0.73
NM_010908	Nfkbib	Nuclear factor of kappa light polypeptide gene enhancer in B-cells inhibitor, beta	0.77
NM_013609	Ngf	Nerve growth factor	2.11
NM_033217	Ngfr	Nerve growth factor receptor (TNFR superfamily, member 16)	1.23
NM_008712	Nos1	Nitric oxide synthase 1, neuronal	0.25
NM_016789	Nptx2	Neuronal pentraxin 2	0.50
NM_010444	Nr4a1	Nuclear receptor subfamily 4, group A, member 1	0.95
NM_008742	Ntf3	Neurotrophin 3	0.75
NM_198190	Ntf5	Neurotrophin 5	3.42
NM_008745	Ntrk2	Neurotrophic tyrosine kinase, receptor, type 2	1.10
NM_021543	Pcdh8	Protocadherin 8	1.37
NM_008837	Pick1	Protein interacting with C kinase 1	0.75
NM_008842	Pim1	Proviral integration site 1	0.24
NM_008872	Plat	Plasminogen activator, tissue	0.85

NM_021280	Plcg1	Phospholipase C, gamma 1	0.77
NM_031868	Ppp1ca	Protein phosphatase 1, catalytic subunit, alpha isoform	0.62
NM_013636	Ppp1cc	Protein phosphatase 1, catalytic subunit, gamma isoform	1.11
NM_026731	Ppp1r14a	Protein phosphatase 1, regulatory (inhibitor) subunit 14A	1.60
NM_019411	Ppp2ca	Protein phosphatase 2 (formerly 2A), catalytic subunit, alpha isoform	0.54
NM_008913	Ppp3ca	Protein phosphatase 3, catalytic subunit, alpha isoform	1.55
NM_011101	Prkca	Protein kinase C, alpha	1.47
NM_011102	Prkcg	Protein kinase C, gamma	1.53
NM_011160	Prkg1	Protein kinase, cGMP-dependent, type I	1.33
NM_009001	Rab3a	RAB3A, member RAS oncogene family	0.77
NM_009045	Rela	V-rel reticuloendotheliosis viral oncogene homolog A (avian)	0.33
NM_011261	Reln	Reelin	0.65
NM_009061	Rgs2	Regulator of G-protein signaling 2	1.22
NM_053075	Rheb	Ras homolog enriched in brain	1.10
NM_019812	Sirt1	Sirtuin 1 (silent mating type information regulation 2, homolog) 1	0.25
NM_020493	Srf	Serum response factor	0.88
NM_177340	Synpo	Synaptopodin	0.64
NM_011593	Timp1	Tissue inhibitor of metalloproteinase 1	1.67
NM_013693	Tnf	Tumor necrosis factor	1.87
NM_011739	Ywhaq	Tyrosine 3-monooxygenase/tryptophan 5-monooxygenase activation protein	2.30

Blue: statistically significant upregulated genes
Red: statistically significant downregulated genes

Table S2. ANTIBODIES

Primary Antibody	Host	Catalogue reference
α-TrkB (80E3)	Rabbit	Cell Signaling #4603
α-pTrkB Tyr816	Rabbit	See Methods
α-Actin	Rabbit	Biorbyt #10033
α-BDNF	Rabbit	Immunological Sciences AB-82598
α-pCreb^{S133}	Rabbit	Millipore 06-519
α-Creb (ChIP)	Rabbit	Millipore 06-863
α-Creb (WB)	Mouse	ThermoFisher #MA1-083
α-Histone H3 (acetyl K9)	Rabbit	Abcam ab4441
α-Alix	Rabbit	Cell signaling #92880
α-CD81	Rabbit	Abcam ab109201
α-Gapdh	Mouse	Abcam ab8245
α-Tubulin	Mouse	Sigma #T6074
α-MAP2	Mouse	Sigma #M4403

Table S3. PRIMERS

Primer sequences used for RT-PCR analyses.

Gene		Primer sequence
nNOS	FW	5'- CTGTGACAACTCTCGATACAACATC -3'
	RV	5'-GTTTGATGAAGGACTCGGTGG-3'
Sirt1	FW	5'-TTTCATTCCTGTGAAAGTGATG-3'
	RV	5'-GTAATAAATCTTTAAGAATTGTTCG-3'
Egr3	FW	5'-TCAACCCCAGTTGTCCTGTCC-3'
	RV	5'-TGTAAACTCCAGACCTTTTGTCGC-3'
RelA	FW	5'-TACCCGAAACTCAACTTCTGTCC-3'
	RV	5'-ACCATGGCTGAGGAAGGGACC-3'
Adam10	FW	5'-CTCTCCATGTAATGACTTCAGAGG-3'
	RV	5'-ATAAATCCAGCCATTAACATGATCAGG-3'
Dlg4	FW	5'-ACCCTATCGCCATCTTCATCCG-3'
	RV	5'-CTGAGAGGTCTTCGATGACACG-3'
Bdnf	FW	5'-TGGCTGACACTTTTGAGCAC-3'
	RV	5'-GTTTGCGGCATCCAGGTAAT-3'
Actin	FW	5'-GTCACCCACACTGTGCCCATCT-3'
	RV	5'-ACCGAGTACTTGCGCTCAGGA-3'

Primer sequences used for ChIP analyses.

Gene		Primer sequence
BDNF promoter I	FW	5'-TCGATTCACGCAGTTGTTCC-3'
	RV	5'-GCACCAGCCGGCTACTGC-3'
BDNF promoter IV	FW	5'-CATGCAATGCCCTGGAACG-3'
	RV	5'-GAGAGCAGTCCTCTCCTCG-3'
nNOS promoter	FW	5'-CCTTTGGTTCTTGAGAGCTTTG-3'
	RV	5'-AGAGAGTGGAGTGGGCACC-3'
Sirt1 promoter	FW	5'-CACGTGACCCGGCGTGTTG-3'
	RV	5'-CCTCTTGCGGAGCGGCTCG-3'
Egr3 promoter	FW	5'-CGCTTCCTGCTTTCTAATGTTCC-3'
	RV	5'-GCTTCCTAGCTAGCTCACTGC-3'

RelA promoter	FW	5’-TGAACTCAGGGTAAAAAGGAATGG-3’
	RV	5’-CGGTGTGATTTTGTCTCAGAGG-3’
Adam10 promoter	FW	5’-CGGCACGCATGCGCATTATCC-3’
	RV	5’-GCTCTTCGCCTGGTCTCAGC-3’
Dlg4 promoter	FW	5’-TTCTGTTCCTGTGACAGACATCC-3’
	RV	5’-TCAGTACTAGGGATCATGTTGCC-3’

Abbreviations: FW, forward; RV, reverse
